# Paraneoplastic Effects of Lung Adenocarcinoma Causing Persistently Elevated Lipase, Amylase, and CA 19-9

**DOI:** 10.14309/crj.0000000000001792

**Published:** 2025-08-01

**Authors:** Lily Lucerne, Saurabh Chawla

**Affiliations:** 1 Emory University School of Medicine, Atlanta, GA; 2 Department of Digestive Diseases, Emory University School of Medicine, Atlanta, GA

**Keywords:** lung adenocarcinoma, paraneoplastic syndrome, elevated lipase, elevated amylase, CA 19-9, misdiagnosed pancreatitis, tumor markers

## Abstract

Lipase, amylase, and carbohydrate antigen 19-9 are associated with pancreatic pathology. We report a rare case of a patient with persistent elevation of these markers due to paraneoplastic effects of lung adenocarcinoma. The patient presented with right upper quadrant pain and had elevated lipase, amylase, and carbohydrate antigen 19-9. Workup was unrevealing for pancreatic pathology but found a lung nodule, later confirmed as adenocarcinoma. The patient underwent chemotherapy, radiation, and immunotherapy. All levels subsequently normalized. This case underscores the importance of considering malignancy in patients with unexplained, persistent elevation of pancreatic enzymes or tumor markers in the absence of clear pancreatic pathology.

## BACKGROUND

Lipase and amylase are enzymes the pancreas produces to break down fats and polysaccharides. Significant elevation in both lipase and amylase, defined as 3 times the upper limit of normal, have high specificity for acute pancreatic inflammation and tend to normalize within a week of the acute episode and, therefore, are recommended as diagnostic markers of acute pancreatitis in the appropriate clinical settings.^1^ Usually, in nonpancreatic etiologies, the elevation of these enzymes is less than 3 times the upper limit of normal. Malignancy may also cause lipase elevation by intrinsic pancreatic causes such as duct obstruction, formation of macrolipases, or tumor mass production/hypersecretion.^2^ Carbohydrate antigen 19-9 (CA 19-9) is a tumor marker, most often associated with pancreatic and other gastrointestinal malignancies but rarely may be elevated in other malignancies, including lung adenocarcinoma.^3^ We report an uncommon presentation of persistent elevation of lipase, amylase, and CA 19-9 due to paraneoplastic effects of lung adenocarcinoma.

## CASE REPORT

The patient is a 72-year-old woman who initially presented with mild right upper quadrant pain for 5 days. The patient reported the pain was intermittent, not associated with eating, and did not improve after bowel movements. The patient had no known tobacco use and had been a social drinker (4 glasses of wine per week)—but no current alcohol use. The initial outpatient workup showed elevated lipase of 942 U/L (normal 11-82 U/L), and the patient was referred to the hospital for further evaluation.

In the emergency department, the patient had normal vital signs, mild tenderness in the right upper quadrant, no rebound tenderness or guarding, and Murphy sign was negative. Blood tests showed elevated levels of lipase (820 U/L) and amylase (283 U/mL, normal 29-103 U/mL). Her creatinine, blood glucose level, and liver function tests were all normal. A contrast-enhanced computed tomography (CT) of the abdomen was unremarkable, and no biliary or pancreatic pathology was visualized. A right upper quadrant ultrasound showed some sludge in the gallbladder with normal-appearing bile ducts. Since her pain had spontaneously subsided, she was discharged with a diagnosis of acute pancreatitis.

**Figure 1.**
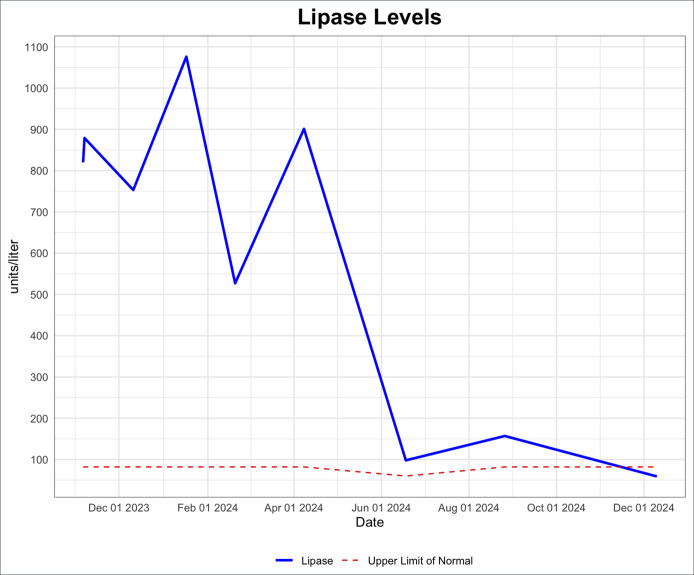
Lipase levels remained greater than 3 times the upper limit of normal before chemotherapy and radiation treatment. Values ranged from 527 to 1076 U/L. Following the completion of chemotherapy/radiation, lipase levels have returned to normal.

**Figure 2.**
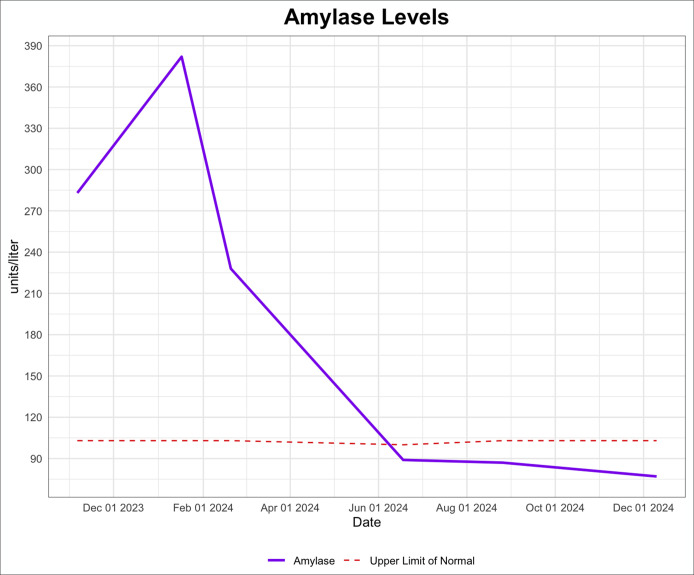
Amylase levels were between 2 and 4 times the upper limit of normal before chemotherapy and radiation treatment. Following completion of chemotherapy/radiation, amylase levels have returned to normal limits.

**Figure 3.**
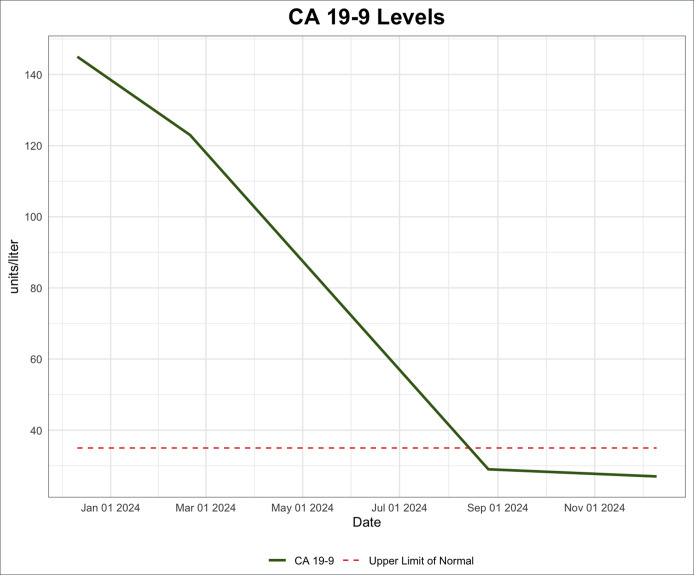
CA 19-9 was measured twice before chemotherapy and radiation. Both times the level was significantly elevated. Following the completion of chemotherapy/radiation, CA 19-9 returned to normal levels. CA 19-9, carbohydrate antigen 19-9.

**Figure 4.**
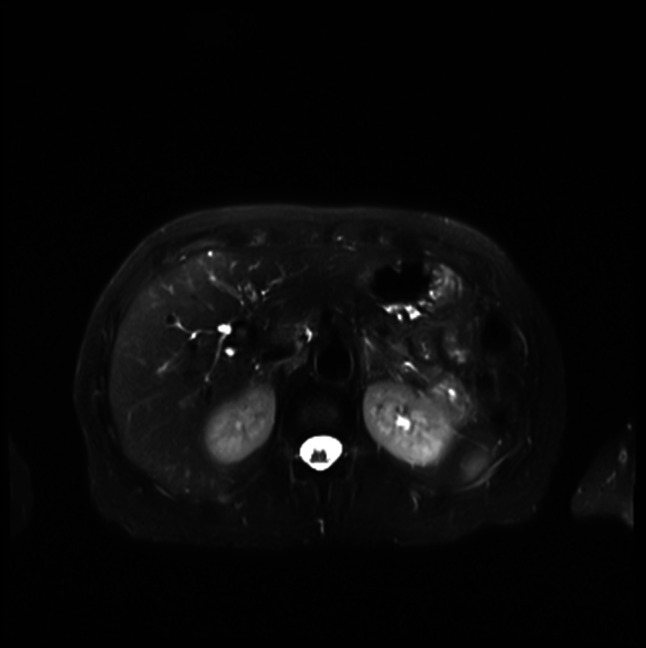
MRI of the abdomen showing cystic lesion at the uncinate process, likely a side branch IPMN. MRI, magnetic resonance imaging.

On outpatient follow-up, lipase (1076 U/L) and amylase (382 U/L) remained elevated although she denied any abdominal pain. Additional testing showed an elevated CA 19-9 level (145 U/L, normal range < 35 U/L). Over the next 3 months, the patient continued to have persistently elevated lipase, amylase, and CA 19-9 levels (Figures 1–3) with no gastrointestinal symptoms, and further workup for pancreatic pathology was initiated. Endoscopic ultrasound showed a 6 mm uncinate side branch dilatation and nonspecific pancreatic parenchymal changes. Abdominal magnetic resonance imaging done to evaluate the pancreas confirmed the endosonographic findings (Figure 4) and incidentally noted indeterminate central right lung nodule and right basilar pleural thickening.

**Figure 5.**
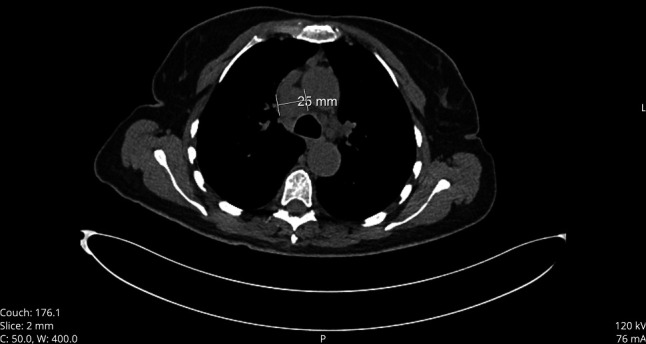
Chest CT showing paratracheal lymphadenopathy. Chest CT from December 14, 2023. CT, computed tomography.

Workup of the lung nodule including a positron emission tomography-CT, bronchoscopy, endobronchial ultrasound, and lymph node biopsy were then performed. Biopsy of the right paratracheal (Figure 5) and subcarinal lymph nodes both showed lung adenocarcinoma consistent with stage 3B lung cancer. Positron emission tomography-CT showed no evidence of distant disease. The patient underwent chemotherapy consisting of paclitaxel and carboplatin, as well as radiation therapy for her lung adenocarcinoma. This was followed by maintenance immunotherapy with durvalumab. The patient's elevated amylase, lipase, and CA 19-9 were noted to downtrend soon after starting chemotherapy and have continued to be within normal limits after completion of chemotherapy for her lung cancer (Figures 1–3).

## DISCUSSION

This case study illustrates the rare occurrence of persistently elevated lipase, amylase, and CA 19-9 levels due to paraneoplastic effects of lung adenocarcinoma. Our patient was initially misdiagnosed with acute pancreatitis due to anchoring bias, despite the patient not meeting two-thirds criteria which delayed the initial workup. Extensive gastrointestinal workup did not show any significant pancreatic masses on magnetic resonance imaging, CT, or endoscopic ultrasound that would explain the persistent elevation. Through continued monitoring, these levels remained elevated, pointing to another cause. These enzymes are typically associated with pancreatic pathology, but they can also indicate paraneoplastic syndromes from malignancy.

A literature review showed 1 other case report of a patient with paraneoplastic elevations of lipase and amylase from small-cell lung cancer.^4^ The patient had metastatic small-cell lung cancer with elevated lipase and amylase. These enzymes were very responsive to chemotherapy treatment—cisplatin and etoposide. Both lipase and amylase returned to normal range values by the end of treatment (6 cycles). That patient's cancer analysis showed lipase and pancreatic isoamylase expression on immunohistochemistry. In a retrospective study that evaluated the prognostic accuracy of serum CA 19-9 in patients with advanced lung adenocarcinoma (stage 3B or 4), pathology samples were analyzed and demonstrated CA 19-9 cancer cell secretion.^5^ Both Casadei Gardini et al^4^ and Soto et al^5^ had immunohistochemical data to support tumor secretion of amylase, lipase, and CA 19-9.

Our case highlights the complexity of diagnosis, the importance of follow-up, and is a unique report of lung adenocarcinoma causing persistently elevated lipase, amylase, and CA 19-9 levels. Furthermore, we were able to demonstrate resolution of these elevated biomarkers with therapy for lung cancer and no subsequent pancreatic pathology on serial imaging and follow-up during the course of our patient's treatment. To our knowledge, there are no other reports showing lung adenocarcinoma causing persistent significant elevation in amylase, lipase, and CA 19-9. Therefore, through this report, we want to emphasize that in patients with persistently elevated lipase, amylase, and CA 19-9 with no clear pancreatic or gastrointestinal cause, a paraneoplastic cause should be investigated.

## DISCLOSURES

Author contributions: L. Lucerne: Contributed to the conception and design of the case report, performed the literature review, acquired clinical data, and drafted the majority of the manuscript. S. Chawla: Conceived the idea for the case report, contributed to the literature review, and provided critical revisions and editorial guidance throughout the writing process.

Financial disclosure: None to report.

Presented at the 2024 Georgia Gastroenterologic and Endoscopic Society Annual Meeting, September 14-15, 2024, Callaway Gardens, Pine Mountain, GA.

Informed consent was obtained for this case report.
